# Class I histone deacetylases catalyze lysine lactylation

**DOI:** 10.1016/j.jbc.2025.110602

**Published:** 2025-08-18

**Authors:** Michelangelo B. Gonzatti, Jordi C.J. Hintzen, Isha Sharma, Mohd. Altaf Najar, Takeshi Tsusaka, Mariola M. Marcinkiewicz, Claudia Veronica Da Silva Crispim, Nathaniel W. Snyder, George M. Burslem, Emily L. Goldberg

**Affiliations:** 1 Department of Physiology, University of California, San Francisco, San Francisco, California, USA; 2 Department of Biochemistry and Biophysics, Perelman School of Medicine, University of Pennsylvania, Philadelphia, Pennsylvania, USA; 3 Aging and Cardiovascular Discovery Center, Department of Cardiovascular Sciences, Lewis Katz School of Medicine, Temple University, Philadelphia, Pennsylvania, USA; 4 Department of Cancer Biology and Epigenetics Institute, Perelman School of Medicine, University of Pennsylvania, Philadelphia, Pennsylvania, USA; 5 Chan-Zuckerberg Biohub, San Francisco, California, USA

**Keywords:** post-translational modification (PTM), histone deacetylase (HDAC), lactic acid, lactate, lysine lactylation, glycolysis, macrophage, protein acylation

## Abstract

Metabolism and post-translational modifications (PTMs) are intrinsically linked, and the number of identified metabolites that can covalently modify proteins continues to increase. This metabolism/PTM crosstalk is especially true for lactate, the product of anaerobic metabolism following glycolysis. Lactate forms an amide bond with the ε-amino group of lysine, a modification known as lysine lactylation or Kla. Multiple independent mechanisms have been proposed in the formation of Kla, including p300/CBP-dependent transfer from lactyl-CoA, a reactive intermediate lactoylglutathione species that non-enzymatically lactylates proteins, and several enzymes are reported to have lactyl transferase capability. We recently discovered that class I histone deacetylases (HDACs) 1, 2, and 3 can all reverse their canonical chemical reaction to catalyze lysine β-hydroxybutyrylation. Here we tested the hypothesis that HDACs can also catalyze Kla formation. Using biochemical, pharmacological, and genetic approaches, we found that HDACs are sufficient to catalyze Kla formation and that HDACs are a major driver of lysine lactylation. Dialysis experiments confirm this is a reversible reaction that depends on lactate concentration. We also directly quantified intracellular lactyl-CoA and found that Kla abundance can be uncoupled from lactyl-CoA levels. Therefore, we propose a model in which the majority of Kla is formed through enzymatic addition of lactate by HDACs 1, 2, and 3.

Modification of proteins by acyl groups, like acetylation, can control a range of protein functions, including localization, structure, and association with binding partners (1). The overarching mechanisms controlling protein acylation are well studied: Acyl groups can be added through enzymatic and non-enzymatic pathways and are removed enzymatically by deacylating enzymes like histone deacetylases (HDACs) and the sirtuins. While acetylation is the best studied protein acylation, other short-chain fatty acids like β-hydroxybutyrate (BHB) (2) and lactate (3) can also covalently modify lysine residues, although the physiological significance of these other acylations remains to be fully understood.

Lactate (ʟ-Lactate) is a short-chain fatty acid produced and secreted by glycolytic cells (4). Lactate concentrations in blood and tissues can range from 0.5 to 20 mM and its production is acutely increased in response to a variety of stimuli, including intense exercise and sepsis (5, 6, 7, 8). In addition to serving as a metabolic fuel, lactate can function as a signaling molecule. For example, lactate can be conjugated to phenylalanine to form *N*-lactoyl-phenylalanine (Lac-Phe) that gets secreted from cells and modifies food intake (9). Lactate can also form a covalent adduct on lysine residues, known as lactylation, that is found on histone and non-histone proteins, and has been reported in many cell types (3, 10, 11, 12, 13, 14, 15).

Like other acylations, lysine ʟ-lactylation (Kla) has been proposed to be formed through a lactyl-CoA-dependent pathway (3, 16). However, lactyl-CoA levels in cells are far less abundant compared to other CoA derivatives, such as acetyl-CoA, propionyl-CoA, and succinyl-CoA (17). Interestingly, several enzymes have been reported to possess lactyl transferase activity and catalyze Kla formation on target proteins (18, 19, 20, 21). HDAC6 was also recently reported to lactylate α-tubulin (22). Although less abundant than ʟ-lactylation, the stereoisomeric ᴅ-lactylation is formed from the non-enzymatic acyl transfer from the glycolytic intermediate S-ᴅ-lactoylglutathione (LGSH) generated in the glyoxylase pathway (23, 24, 25). Therefore, multiple non-redundant pathways may contribute to overall intracellular Kla levels.

We recently discovered that class I HDACs 1, 2, and 3 are reversible and catalyze lysine β-hydoxybutyrylation (Kbhb) through a condensation reaction between the BHB carboxylic acid and free amine on lysine residues (26). In this new HDAC-catalyzed mechanism of protein acylation, we ruled out a requirement for an activated intermediate like BHB-CoA. We also showed that the same active site residues were required for both deacetylation and β-hydroxybutyrylation, consistent with our model that HDACs 1, 2, and 3 are reversible enzymes that catalyze the addition or removal of acyl chains depending on amenable substrate concentration. Of note, our model of HDAC-catalyzed protein acylation has been independently verified by others for lysine sorbylation (27, 28). As we and others have reported that HDACs 1, 2, and 3 can de-lactylate proteins (29, 30), we hypothesized that, like Kbhb formation, HDACs also contribute to Kla formation through a similar reversible mechanism. Using biochemistry, molecular biology, and mass spectrometry, we confirmed HDACs 1, 2, and 3 are capable of directly catalyzing the formation of Kla in a recombinant reconstitution assay and in live cells. Furthermore, we used a lysine protection assay with fluorogenic peptide to measure HDAC-catalyzed Kla kinetics and confirmed that this reaction occurs at physiological lactate concentrations. Altogether, our data highlight the broad contributions of HDAC-catalyzed lysine acylation and directly link metabolism to protein modifications.

## Results

### HDACs 1, 2, and 3 catalyze lysine lactylation

**Figure 1:**
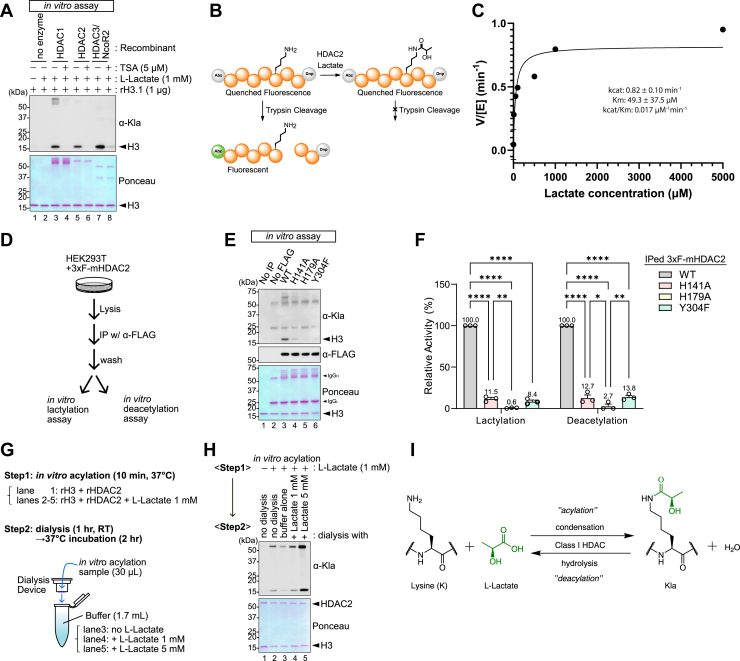
**HDACs 1, 2, and 3 catalyze lysine lactylation *in vitro*.***A*, *in vitro* lysine lactylation assay with recombinant HDAC1 (rHDAC1), rHDAC2, or rHDAC3/NcoR2 and histone H3 (rH3) in the presence of 1 mM ʟ-Lactate with or without 5 μM TSA. Reactions were performed at 37 °C for 30 min. Protein loading was visualized by ponceau S staining, and Kla was detected by Western blot. Representative of two independent experiments. *B–C*, HDAC-catalyzed Kla formation kinetics were quantified in a lysine protection assay. *B*, schematic of experimental design. *C*, calculated reaction rate of lactylated H3K9 peptide. *D*–*F*, HDAC2 KO HEK293T cells expressing 3xFLAG-mHDAC2 were used for immunoprecipitation (IP) with α-FLAG antibody. The immunoprecipitants were used for *in vitro* lysine lactylation and deacetylation assays. Parental HDAC2 KO HEK293T lacking transgene expression were used as control cells for IP. *D*, schematic of experimental workflow. *E*, Western blots for indicated targets of *in vitro* lysine lactylation using immunoprecipitated 3xF-mHDAC2 WT or the indicated mutants. All lanes also contained L-lactate (5 mM) and rH3 (1 μg). Representative of three independent experiments. *F*, quantification of lysine lactylation and deacetylation activity for each mutant. Data represent mean ± SEM, relative to WT 3xF-mHDAC2, from three independent experiments. Each symbol represents an individual experiment. *G*, Schematic of the experimental workflow to assess lactate concentration-dependent reversibility of HDAC using a dialysis system. *H*, Western blot showing Kla levels before and after dialysis with different ʟ-Lactate concentrations. Representative of two independent experiments. *I*, proposed model of the forward and reverse reactions catalyzed by class I HDACs. ∗*p* < 0.05, ∗∗*p* < 0.01, ∗∗∗∗*p* < 0.0001.

We previously used an *in vitro* reconstitution assay to show that recombinant HDAC2 (rHDAC2) can biochemically catalyze lysine acylation by short-chain fatty acids (26). We confirmed these results with lactate and verified that HDACs 1, 2, and 3 are each capable of this biochemical reaction and this could be inhibited by coincubation with the pan-HDAC active site inhibitor Trichostatin A (TSA) (Fig. 1*A*). We used liquid chromatography tandem mass spectrometry (LC-MS/MS) to validate the Western blot data and identified histone H3-tail lysine residues K9, K14, K18, K23, K27, K36, K37, and globular lysines K56, K79, and K122 as lactylated sites by rHDAC2 in this *in vitro* assay (Fig. S1, *A* and *B*). Notably, unlike the proposed p300-dependent lactyl transferase mechanism, we confirmed that HDACs can catalyze lysine lactylation with both ᴅ- and ʟ-lactate. These data indicate that, like Kbhb, HDAC enzymatic activity is sufficient for Kla formation. We developed a lysine protection assay to measure HDAC-catalyzed Kla formation kinetics using a synthesized fluorogenic peptide sequence selected based on the Kla LC-MS/MS analysis (Figs. 1, *B*, *C* and S1, *C*, *D*) (30, 31). This experiment revealed kcat = 0.82 ± 0.10 min^−1^ and Km = 49.3 ± 37.5 μM, confirming HDAC-catalyzed Kla formation is biochemically possible at physiological lactate concentrations.

We next tested the importance of key HDAC active site residues in lysine lactylation. We used HDAC2 knockout HEK293T cell lines expressing 3xFLAG-tagged mouse HDAC2 (mHDAC2) wild-type (WT) and individual point mutants H141A, H179A, and Y304F. These mutants each have a single substitution of residues required for deacetylation that we previously showed are also required for lysine β-hydroxybutyrylation (26, 32). We purified these WT or mutant HDAC2 proteins using anti-FLAG conjugated beads and performed our *in vitro* lactylation assay with rH3 as a model substrate. The WT 3xF-mHDAC2 showed the strongest lysine lactylation activity toward rH3, while all three mutants decreased the lactylation activity as expected (Fig. 1, *D*–*F*). To test the reversibility of the enzymatic activity of rHDAC2 toward lysine lactylation, we used a dialysis system in combination with our *in vitro* lactylation assay to manipulate lactate concentrations (Fig. 1, *G* and *H*). When lactate was removed from the reaction conditions by dialysis, rHDAC2 removed the lactyl modification from H3, consistent with a previous study (29). In contrast, lysine lactylation levels were retained or even increased when dialysis was performed in buffers containing the same or higher concentrations of lactate, respectively. Taken together, these data are consistent with a model in which the HDAC2 catalysis is reversible, enabling it to either add or remove lactate from lysine residues, depending on the concentration of lactate (Fig. 1*I*).

### HDACs are required for Kla formation in cells

**Figure 2:**
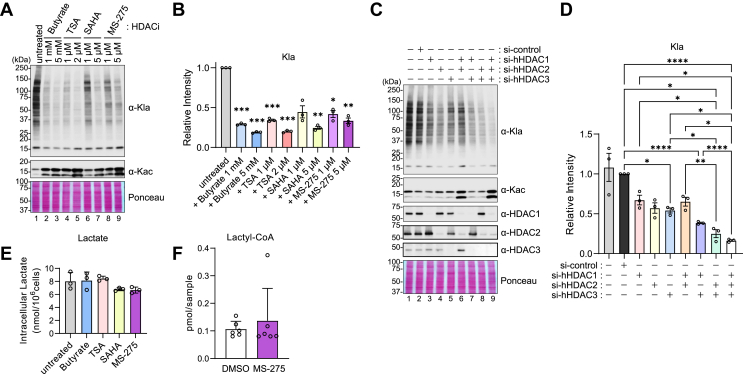
**Catalytic activities of HDAC1, 2, and 3 are required for Kla formation in cells.***A–B*, HEK293T cells were treated with the indicated HDAC inhibitors at the specified concentrations for 24 h. *A*, representative western blots showing anti-Kla and anti-Kac signals. *B*, quantification of Kla levels relative to untreated control, normalized to ponceau S staining. Data represent mean ± SEM from three independent experiments. Each symbol represents an individual experiment. Statistical significance was determined by one-way ANOVA followed by Dunnett’s correction for multiple comparisons. *C–D*, HEK293T cells were transfected with siRNAs targeting the indicated genes. *C*, representative western blots for the indicated targets. *D*, quantification of Kla levels relative to si-control, normalized by ponceau S staining. Data represent mean ± SEM of three independent experiments. Each symbol represents an individual experiment. Statistical significance was determined by one-way ANOVA followed by Tukey’s test for multiple comparisons. *E*, quantification of intracellular lactate concentrations in HEK293T cells treated with HDAC inhibitors: Butyrate (5 mM), TSA (1 μM), SAHA (5 μM), MS-275 (5 μM). Data represent mean ± SD from three technical triplicates of a single experiment. Each symbol represents a technical replicate. *F*, quantification of intracellular lactyl-CoA concentrations in HEK293T cells treated with MS-275 (5 μM). Data are represented as mean ± SD from six technical triplicates of a single experiment, each symbol represents a technical replicate. ∗*p* < 0.05, ∗∗*p* < 0.01, ∗∗∗*p* < 0.001, ∗∗∗∗*p* < 0.0001.

Next, we investigated the relevance of HDAC-catalyzed lysine lactylation in live cells. We treated HEK293T cells with several HDAC inhibitors (HDACi), including butyrate, TSA, SAHA, and MS-275. Of note, unlike Kbhb, which requires exogenous treatment of BHB in non-ketogenic cells, HEK293T cells basally contain many Kla-modified proteins, presumably due to their high metabolic and proliferative nature. All the HDAC inhibitors broadly decreased basal Kla levels and increased lysine acetylation (Kac) as expected (Figs. 2, *A*, *B* and S2). Next, we knocked down HDAC1, HDAC2, and HDAC3 in varying combinations in HEK293T cells to further evaluate the role of HDACs in intracellular Kla formation. Each single knockdown showed a similar reduction (approximately ∼50%, lanes 3–5) in Kla levels, and the triple knockdown showed the strongest effect on Kla levels (lane 9), suggesting HDACs 1, 2, and 3 redundantly contribute to Kla formation in cells (Fig. 2, *C* and *D*). Of note, these data contradict a prior similar experiment (29), but we believe this is due to differences in HDAC knockdown efficiency between our studies, with the HDAC knockdown being more complete in our current experiments, which is evident by the expected increased histone Kac in our blots. We also confirmed that HDACi treatment did not affect intracellular lactate or lactyl-CoA concentrations (Fig. 2, *E* and *F*), indicating that class I HDACs directly control Kla levels, rather than indirectly controlling substrate availability to modify Kla abundance.

### Kla formation is regulated by glucose metabolism

**Figure 3:**
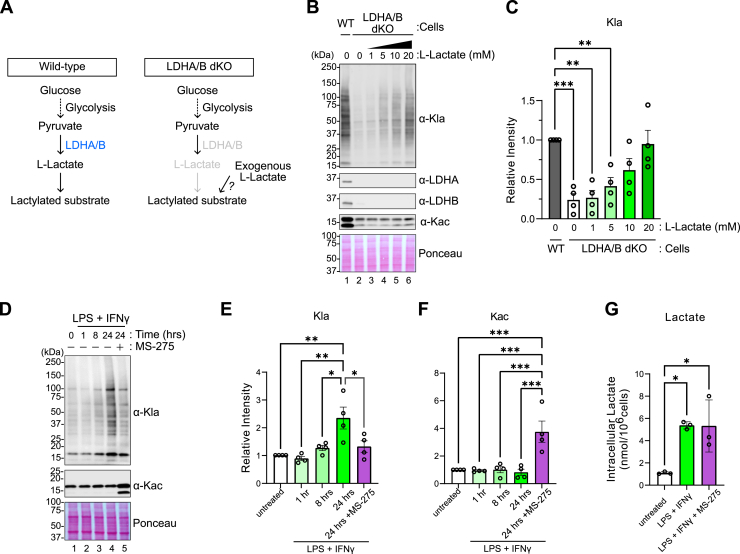
**Kla formation is regulated by glucose metabolism.***A*, schematic illustrating anticipated Kla levels in wild-type (WT) and established LDHA/LDHB double knock-out (LDHA/B dKO) cells. *B-C*, LDHA/B dKO HEK293T cells were treated with ʟ-Lactate at the indicated concentrations for 24 h. *B*, representative western blots for the indicated targets. *C*, quantification of Kla levels relative to untreated control, normalized to ponceau S staining. Data are represented as mean ± SEM from four independent experiments. Each symbol represents an individual experiment. Statistical significance was determined by one-way ANOVA followed by Dunnett’s correction for multiple comparisons. *D–G*, bone marrow-derived macrophages (BMDM) were stimulated with LPS (5 ng/ml) and IFNγ (12 ng/ml) for the indicated time points. MS-275 (0.5 μM) was added 8 h after LPS + IFNγ stimulation, resulting in 16 h of inhibition (from T8 to T24). *D*, representative western blots showing anti-Kla and anti-Kac signals. *E*, quantification of Kla levels relative to untreated control, normalized to ponceau S staining. *F*, quantification of Kac levels relative to untreated control, normalized to ponceau S staining. Data represent mean ± SEM from two independent experiments that each contained two biological replicates; each symbol represents a biological replicate. Statistical significance was determined by one-way ANOVA followed by Tukey’s correction for multiple comparisons. *G*, quantification of intracellular lactate concentrations in BMDM after 24 h of stimulation with LPS (5 ng/ml) and IFNγ (12 ng/ml), with or without MS-275 (0.5 μM). Data represent mean ± SD from a single experiment with three biological replicates. Each symbol represents a biological replicate. Statistical significance was determined by one-way ANOVA followed by Dunnett’s correction for multiple comparisons. ∗*p* < 0.05, ∗∗*p* < 0.01, ∗∗∗*p* < 0.001.

Because glycolysis-derived ʟ-lactate and glyoxylase-derived ᴅ-lactate have both been implicated in Kla formation and HDACs can use both isomers, we examined the relative importance of each of these isomers in HDAC-catalyzed Kla in cells. First, we generated LDHA/LDHB double knock-out (LDHA/B dKO) HEK293T cells to disrupt ʟ-lactate production (Figs. 3, *A*–*C*, S3, *A* and *B*). We observed a striking reduction in global Kla modifications in the LDHA/B dKO cells that were rescued by exogenous ʟ-Lactate supplementation. We confirmed that Kla rescue in the LDHA/B dKO cells also matched the expected subcellular distribution, with most Kla-modified proteins found in the nuclear and chromatin fractions, followed by the cytoplasm, in agreement with a prior study (33). This distribution of Kla modifications matches HDAC 1, 2, and 3 fractionation (Fig. S3*C*). To further demonstrate the dependence of Kla on class I HDACs, we used native LDHA/B-dKO cell lysates in our *in vitro* reconstitution assay with 10 mM ʟ-lactate and increasing concentrations of rHDAC2 (Fig. S3*D*). In this setting of high lactate levels, increasing the amount of HDAC2 led to higher Kla levels. We became aware of a preprint demonstrating that the pan-Kla antibody also detects carboxyethyl-lysine (Kce) modifications (34). Using their same *in vitro* system, we confirmed that at high *in vitro* concentrations, the pan-Kla antibody does react with Kce-modified BSA (Fig. S4, *A* and *B*). Next, we knocked down GLO1 with siRNA, which would lead to the accumulation of methylglyoxal (MGO) and thus increase Kce and decrease ᴅ-lactyl-lysine (24, 25) but should have no impact on ʟ-lactate or ʟ-lactyl-lysine. GLO1 knockdown did not change global Kla levels as detected with the pan-anti-Kla antibody that recognizes ʟ-lactyl-lysine (Fig. S4, *C*–*E*). Moreover, when we used a pan-Kce antibody to detect Kce-modified proteins, in comparison to the Kce-modified BSA, only a very weak signal was detected in the cell extract samples, with no apparent change upon GLO1 knockdown, suggesting that this PTM might only be present at very low levels in cells. (Fig. S4*F*). Of note, we would expect our LDHA/B dKO to possibly also increase Kce modifications due to the buildup of pathway intermediates, but we observed no Kce-modified proteins in these cells either. These data strongly support our conclusion that the pan-Kla antibody is detecting lactylated proteins in our experiments and indicate that the lactate derives from glycolysis.

Lactate is a byproduct of glycolysis and is secreted from highly glycolytic cells. As such, we cultured HEK293T cells under no, low (1 g/L), and high (5 g/L) glucose conditions for 24 h and observed that Kla modification across all proteins correlated with glucose concentration and consequent lactate concentration (Fig. S5, *A*–*C*). We also observed reduced global lysine lactylation in oxamate (OXA) or dichloroacetate (DCA)-treated cells (Fig. S5, *D*–*F*), both of which can lower lactate production. We also confirmed that co-treating cells with TSA to inhibit HDAC enzymatic activity blocked the glucose-driven Kla accumulation (Fig. S5, *G* and *H*), supporting our model that glycolysis-derived lactate is used in HDAC-catalyzed Kla formation. Notably, unlike 20 to 24 h of TSA treatment, 4 h of TSA treatment did not change Kla levels. This result agrees with prior work and is likely due to the inhibition of Kla removal by HDACs (29). Because all our work thus far was in glycolytic proliferating cells, we wanted to test the role of HDACs in acute Kla formation. For these experiments, we used a model of primary murine bone marrow-derived macrophages (BMDM). Activation of macrophages with the toll-like receptor 4 (TLR4) ligand lipopolysaccharide (LPS) leads to acute upregulation of glycolysis and lactate secretion. In agreement with a prior study (3), we confirmed that LPS or LPS + interferon (IFN) γ activation also leads to Kla accumulation, and importantly, this was blocked when BMDM were co-treated with the HDAC inhibitors MS-275 or butyrate during LPS treatment (Figs. 3, *D*–*F* and S5, *I*–*K*). We also noted that LPS or LPS + IFNγ stimulation induced global lysine lactylation, not just on histones, and both were prevented by MS-275 or butyrate treatment. We also confirmed that MS-275 treatment did not affect lactate production in activated BMDM (Fig. 3*G*). These results show that glycolytically activated macrophages rely on HDAC activity to promote Kla formation.

### Histone acyltransferases do not control global Kla in untreated proliferating HEK293T cells

Because lactate rescued Kla formation in LDHA/B-dKO cells but could be uncoupled from Kla formation in BMDM, we tested if an alternative lactate-dependent pathway controls global Kla levels in HEK293T cells. Recently, the alanyl-tRNA synthetases, AARS1 and AARS2, were reported to act as lactyl-transferases using lactate and ATP (19, 20, 21) (Fig. S6*A*). However, this function of AARS enzymes was studied in the context of glucose starvation or largely focused on lactylation of specific target proteins. To compare the relative contribution of AARS enzymes towards global basal levels of Kla in cells, we used siRNA to knockdown *AARS1* and *AARS2*. However, the knockdown of either enzyme did not alter Kla levels (Fig. S6, *B*–*E*), suggesting the AARS pathway is also not required for global lysine lactylation.

**Figure 4:**
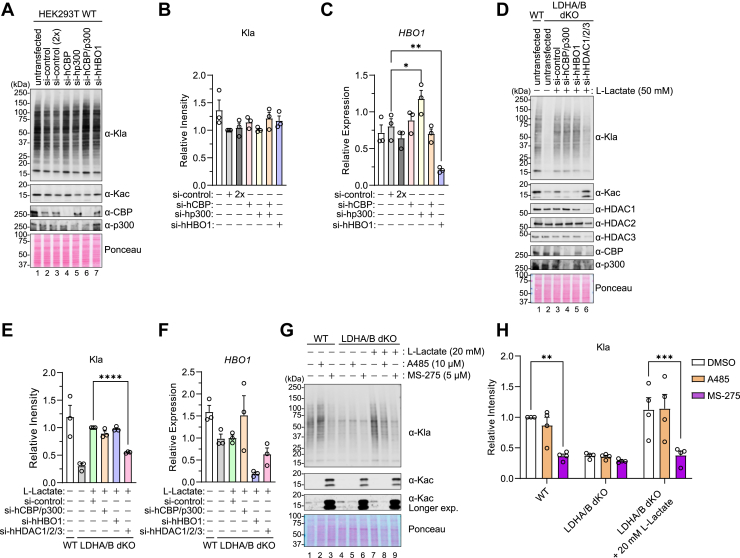
**Acyl transferases are not required for Kla formation.***A–C*, HEK293T cells were transfected with siRNA targeting the indicated genes. *A*, representative western blots for the indicated targets. *B*, quantification of Kla levels relative to si-control. Signals were normalized to ponceau S staining. *C*, quantification of *HBO1* mRNA expression relative to si-control, normalized by *RPL13A*. *B* and *C*, data represent mean ± SEM from three independent experiments and each symbol represents an individual experiment. Statistical significance was determined by one-way ANOVA followed by Dunnett’s correction for multiple comparisons. *D–F*, LDHA/B dKO HEK293T cells were transfected with siRNAs targeting the indicated genes and treated with ʟ-Lactate 50 mM for 8 h. WT HEK293T and untransfected LDHA/B dKO cells were used as controls. *D*, representative western blots for the indicated targets. *E*, quantification of Kla levels relative to si-control, normalized to ponceau S staining. *F*, quantification of *HBO1* mRNA expression relative to si-control, normalized by *RPL13A*. For (*E*, *F*) data are represented as mean ± SEM of three independent experiments and each symbol represents an individual experiment. Statistical significance was determined by one-way ANOVA followed by Dunnett’s correction for multiple comparisons. *G-H*, LDHA/B dKO HEK293T cells were treated with ʟ-Lactate (20 mM) and either A485 (10 μM) or MS-275 (5 μM) for 24 h. DMSO was used as vehicle control for HDAC inhibitors. WT HEK293T cells not treated with ʟ-Lactate were used as additional controls. *G*, representative western blots for the indicated targets. *H*, quantification of Kla levels relative to DMSO-treated controls for each condition, normalized to ponceau S staining. Data represent mean ± SEM from four independent experiments, each symbol represents an individual experiment. Statistical significance was determined by one-way ANOVA followed by Dunnett’s correction for multiple comparisons. ∗*p* < 0.05, ∗∗*p* < 0.01, ∗∗∗*p* < 0.001, ∗∗∗∗*p* < 0.0001.

Because lactate is also the obligatory precursor of lactyl-CoA, we tested the roles of reported lactyl-CoA synthetases in lysine lactylation (Fig. S7*A*). Two enzymes have been reported to be lactyl-CoA synthetases: ACSS2 (acetyl-CoA synthetase 2) and the SUCLG2 (succinate-CoA ligase GDP-forming subunit beta) subunit of guanosine triphosphate-specific SCS (GTPSCS) (35, 36). When we used siRNA to knock down *ACSS2* and *SUCLG2* in HEK293T cells, we observed a modest but reproducible decrease in Kla after *ACSS2* knockdown, but no effect after *SUCLG2* knockdown (Fig. S7, *B*–*E*). Unexpectedly, targeting ACSS2 and SUCLG2 did not change intracellular lactyl-CoA levels (Fig. S6*F*). Next, we tested the putative lactyl-transferases CBP, p300, and the recently reported lactyltransferase HBO1 (18). When we knocked down *CBP*, *p300*, and *HBO1* with siRNA in wild-type HEK293T cells, we observed no change in Kla levels (Fig. 4, *A*–*C*). We used the same siRNA approach in LDHA/B dKO cells that were rescued with lactate and found that knockdown of HDACs1-3, but not CBP + p300 or HBO1, inhibited Kla formation (Fig. 4, *D*–*F*). We used a complementary pharmacological approach to test if Kla rescue in lactate-supplemented LDHA/B-dKO cells was p300/CBP-dependent or HDAC-dependent (Fig. 4, *G* and *H*). When LDHA/B-dKO cells were treated with exogenous ʟ-lactate to restore Kla levels, the p300/CBP inhibitor A485 did not prevent Kla formation, but HDAC1-3 inhibitor MS-275 still blocked Kla formation. In agreement with prior work, these data confirm that a p300/CBP-dependent acyl transfer from lactyl-CoA is not the primary mechanism of global Kla formation (35).

## Discussion

Our data highlight the important role of class I HDACs in Kla formation. We and others routinely see many lactylated proteins in untreated proliferating cultured cells, and we hypothesize this is due to increased lactate concentrations in the culture media over time. Based on our knockdown experiments of acyl transferases p300, CBP, and HBO1, our data also suggest that a lactyl-CoA-dependent mechanism is not the primary regulator of global Kla formation at basal levels (Fig. 4). Importantly, p300 and CBP were also previously shown not to regulate histone Kla under EGF or hypoxia conditions (35). Rather, our kinetics and thermodynamics measurements of HDAC-catalyzed Kla reveal that this pathway is more likely to occur at lower physiological lactate concentrations. We found that HDAC2-catalyzed lactylation with lactate as the substrate occurs at a rate of kcat = 0.82 ± 0.10 min^−1^ and at a Km = 49.3 ± 37.5 μM (Fig. 1*C*). Although KAT2A (also known as GCN2)-catalyzed Kla formation using lactyl-CoA as a substrate has a 100-fold lower Km (Km = 0.4890 μM) (35), intracellular lactyl-CoA levels are 0.01 pmol/10^6^ cells (Fig. 2*F*, (17)), whereas our lactate quantification revealed roughly 800,000-fold higher lactate concentrations (∼8 nmol/10^6^ cells, Fig. 2*E*). These metabolite concentrations mean that at physiological levels, the HDAC-catalyzed Kla formation with lactate is strongly favored. Furthermore, an enzyme reported to synthesize lactyl-CoA, GTPSCS/SUCLG2 requires much higher levels of lactate (Km = 15.32 ± 1.28 mM) (36) and may therefore only generate appreciable amounts of lactyl-CoA under extreme conditions like intense exercise, sepsis, or cancer. Interestingly, most of the lactyl-CoA experiments by Liu *et al.*, were performed in U87 glioma cells (36), which could be an important difference and explain why GTPSCS/SUCLG2 knockdown in HEK293T cells in our study had no effect on lactyl-CoA or Kla levels (Fig. S7). Likewise, the reported Km for AARS1 to generate lactyl-AMP to transfer lactate to lysine has Km = 36 ± 7.09 mM, again making this pathway unlikely to occur at healthy lactate levels (20). Based on the reported reaction kinetics so far, we conclude that reversible HDAC activity is a major driver of Kla formation, but additional pathways can operate under different physiological conditions or in cells with specific metabolic profiles.

This result is in agreement with our recent discovery that HDACs 1, 2, and 3 catalyze Kbhb formation (26). We expect HDACs might utilize other short-chain fatty acids for known and yet-to-be-discovered lysine acylations, like the recently reported sorbylation (28) and crotonylation (37) and that this will depend on whether those short-chain fatty acids can achieve the requisite concentrations through metabolic or dietary exposure. Excitingly, HDAC6 was reported to lactylate tubulin, also exposing the possibility that this mechanism may apply more broadly beyond the class I HDACs that we studied here. While the classical understanding of lysine acylation most often involves high-energy metabolites like acyl-CoAs that mediate acyl group transfer, we and others report independent alternative lysine lactylation mechanisms, including (i) the condensation reaction catalyzed by HDACs, (ii) the transfer catalyzed by AARS using ATP and lactate (19, 20, 21), and (iii) non-enzymatic reaction *via* lactoyl-glutathione (24, 25). All of these results underscore how metabolism is directly linked to protein modifications in a way that was not previously appreciated. Future studies examining how these different mechanisms are balanced in different cell types or metabolic conditions, and how these different pathways may compete or target different proteins for lactylation, will enhance our collective understanding of protein acylation.

One of the greatest limitations of our work is that we have been unable to uncouple the forward and reverse HDAC activity to study lactylation in the context of normal HDAC deacetylase activity. This caveat raises the question of whether the changes in Kla simply reflect changes in Kac, such as HDAC inhibition blocked Kla formation while simultaneously increasing Kac levels (Fig. 2, *A* and *C*). Although we cannot conclusively address this question until we have better tools, there are several pieces of data that suggest Kla can be regulated independently of Kac. First, all the *in vitro* reconstitution assays are performed in the absence of acetylation and demonstrate that HDAC-catalyzed Kla formation is an independent function. Second, when we knocked down CBP/p300 (Fig. 4, *A* and *D*), which decreased histone Kac as expected, but did not show a consequential increase in Kla, which one might have expected if Kla levels were purely dependent on Kac levels. In addition, when we rescued Kla levels in LDHA/B dKO cells by treating with exogenous lactate (Fig. 3*B*), the WT, dKO, and dKO + lactate conditions all have similar Kac, indicating Kla can still be newly formed in the presence of homeostatic Kac levels. Likewise, when we cultured cells in the presence of TSA and increasing glucose concentrations, 4 h of TSA treatment (lanes 7–9) increased Kac to similar levels in the presence of increasing Kla levels (Fig. S5*G*). These data confirm that Kla also does not impede Kac formation and indicate that Kla and Kac are not purely exchanged with one another. Whether Kla impacts protein modifications on nearby amino acids remains unknown. A recent analysis comparing all reported Kac and Kla positions found that only 36.7% of the reported Kla modification sites in the literature were previously identified as Kac sites, similar to what we previously found in a similar analysis strategy for Kbhb (26, 33). Overall, these data are consistent with our model that independent enzymes are responsible for the deposition of these different lysine modifications and suggest that the formation of Kla, which we expect to be far lower in abundance and stoichiometry compared to histone Kac (38, 39), may not depend on overall Kac, although this remains to be empirically tested at global and at specific positions that might be modified by both acylation marks.

An open question remains as to the biological function of HDAC-catalyzed lysine lactylation. We have uncovered some of the biochemical parameters of this pathway and confirmed that it operates in cells, but the purpose of this mechanism and under what conditions this pathway is activated remain unknown. Similarly perplexing is that many studies have reported transcriptional activation functions of Kla while HDACs are generally considered transcriptional repressors. If the distribution of Kla is broad but at very low stoichiometric levels even at basal levels, as we suspect, this could have negligible impact or is part of the noise we all observe in standard cell culture. But the presence of so many different possible pathways for Kla formation could become important in different diseases or physiological states, when a new pathway might operate. Therefore, comparing the target specificity of all these different pathways remains a top priority for understanding the significance of Kla in different metabolic conditions. How HDACs target specific proteins for acylation, and how this might depend on the fatty acid acyl group, remains unknown. Given that this pathway operates for multiple substrates and has been demonstrated for multiple HDACs, we propose that HDAC-catalyzed lysine acylation has broader importance in nutrient sensing and linking cell signaling to the metabolic state.

## Experimental procedures

### Mice

All mice were housed and maintained in a specific pathogen-free animal facility at the University of California. All animal protocols were approved by the UCSF Institutional Animal Care and Use Committee. Adult male and female mice (2- to 4-month-old) were used for BMDM experiments. C57BL6/J mice were obtained from the Jackson Laboratory (stock #000664).

### Cell culture

HEK293T cells were generally cultured in high glucose (4.5 g/L ≈ 25 mM) DMEM (Gibco, 10569044) supplemented with 10% fetal bovine serum (FBS) and 1x antibiotic-antimycotic (Gibco, 15240062) in a 37 °C incubator with 5% CO_2_. For glucose starvation/supplementation experiments, cells were cultured in no-glucose DMEM (Gibco, 11966025) supplemented with 10% dialyzed FBS (Gibco, A3382001), 1% GlutaMax (Gibco, 35050-061), 1 mM Sodium Pyruvate (Gibco, 11360-070), 1x antibiotic-antimycotic (Gibco, 15240062). D-glucose (G7021, Silgma-Aldrich) was dissolved in Ultrapure Distilled Water (10977015, Invitrogen) at 100 g/L as a stock, sterilized by filtration, and added to the medium to achieve the indicated glucose concentration. HDAC2 KO HEK293T cells and 3xFLAG-mHDAC2 wild-type (WT) or mutant-expressing cell lines were generated previously (26). Cells were treated as indicated in the respective figure legends with the following reagents: sodium L-Lactate (71718, Sigma-Aldrich), sodium butyrate (B5887, Sigma-Aldrich), Trichostatin A (TSA, T8552, Sigma-Aldrich), Suberoylanilide Hydroxamic Acid (SAHA, 10009929, Cayman), MS-275 (14043, Active Motif), A485 (63875, Tocris), sodium dichloroacetate (DCA, S8615, Selleckchem), and sodium oxamate (OXA, S6871, Selleckchem).

Bone marrow-derived macrophages (BMDM) were established and cultured in RPMI supplemented with 10% FBS, 1x antibiotic-antimycotic, and 20 ng/ml MCSF (Peprotech #315-02), for 7 days. For stimulation, BMDMs were seeded at 1 × 10^6^ cells/ml in a 24-well plate. After an over-night incubation, pre-warmed (37 °C) medium containing 6x stimulants was added to the culture to achieve a final concentration of LPS (O11:B4,1 μg/ml) or LPS (5 ng/ml) + IFNγ (12 ng/ml, eBioscience #14-8311-63).

### siRNA transfection

Cells were transfected with siRNA as described previously (26). Briefly, siRNA at 10 nM, Opti-MEM, and RNAiMax (Life Technology) were mixed in each well of a 6-well dish. After a 10-min incubation, cell suspensions (4 × 10^5^ cells/well) were added to each well, and antibiotic-free medium was added to bring the culture volume to 2 ml. After 48 h, cells were harvested, and the siRNA transfection was repeated once more. The next day, the medium was replaced with pre-warmed (37 °C) fresh medium, and after at least 4 h of incubation, cells were collected for Western blot or qPCR analysis. The following Silencer Select siRNA (Thermo Fisher Scientific) were used: s73 (HDAC1, validated), s6495 (HDAC2, validated), s16876 (HDAC3, validated), s842 (AARS1, validated), s33175 and s33177 (AARS2), s31745 and s31746 (ACSS2), s16775 and s16776 (SUCLG2), and s5825 (GLO1, validated), s3495 (CBP, validated), s4697 and s534247 (p300), and s255 (HBO1, validated). Efficient knock-downs for each gene were validated by qPCR or Western blot analysis. For AARS2, ACSS2, SUCLG2, and p300, two siRNAs were mixed and used for efficient knockdown and to avoid off-target effects.

### Plasmids

Oligo DNA that contained each gRNA sequence encoding human LDHA or LDHB was inserted into lentiGuide-Puro vector (52963, Addgene) according to the Addgene resource information. The following gRNA sequences were used: g-hLDHA#1 (ACAACTGTAATCTTATTCTG), g-hLDHA#2 (AGCCGTGATAATGACCAGCT), g-hLDHA#4 (GGGGAACATGGAGATTCCAG), g-hLDHB#1 (TACATCCACTTCCAATCACG), and g-hLDHB#2 (GGACTGTACTTGACGATCTG). All plasmids used in this study were confirmed by Sanger sequencing.

### CRISPR/Cas9-mediated gene knockout in cells

Cas9-expressing HEK293T cells were previously established (26). Cas9+ HEK293T cells were reverse-transfected with each gRNA-expressing vector using Lipofectamine 3000 and selected with puromycin at 2 μg/ml and blastcidin at 10 μg/ml. After confirming the efficient reduction of target protein in bulk cells by Western blot, single clones for each gRNA were cloned and tested. For LDHA KO, we isolated five clones and used the LDHA KO#1 cl.2 for g-hLDHB transfection. For LDHA/B dKO, we isolated five clones and used the LDHA/B dKO #2 cl.4 for Figures 3 and 4.

### Western blot analysis

Cells were harvested after trypsinization and quenching with medium, washed once with PBS, and lysed with RIPA buffer (Thermo Scientific) supplemented with protease inhibitors (Thermo Scientific). The cell lysates were sonicated using a Branson Sonifier 450 until the viscosity of lysates disappeared and then centrifuged at 14,000 rpm (17,968×*g*) at 4 °C for 5 min to clarify cell lysates. Protein was quantified with the DC Protein Assay Kit (BioRad Laboratories). SDS-PAGE samples were prepared by mixing clarified lysates with 4× Laemmli buffer/10% β-mercaptoethanol (BME) (BioRad Laboratories) and then boiled at 95 °C for 5 min. Equivalent amounts of protein were resolved by SDS-PAGE and then transferred to a nitrocellulose membrane using the Trans-Blot Turbo Transfer system (BioRad Laboratories). Membranes were stained with Ponceaus S, blocked with 5% milk/TBS-T (Tris Buffered Saline/0.1% Tween-20) for 30 to 60 min, and incubated with primary antibodies, followed by secondary HRP-conjugated antibodies. Blots were developed using a chemiluminescent substrate (SuperSignal West Pico PLUS or SuperSignal West Femto Maximum Sensitivity Substrate; Thermo Scientific) and imaged with an Azure 300 (Azure Biosystems). Ponceau S staining was used to confirm equal total protein loading across samples.

### Antibodies

The following antibodies were used in this study: anti-Kac antibody mix (1:1000-3000, CST, 9814S), anti-Kla (1:1000, PTM Biolabs, PTM-1401RM), anti-Kce (1:1000, PTM Biolabs, PTM-1701RM), anti-HDAC1 (1:1000, CST, 5356), anti-HDAC2 (1:1000, CST, 5113), anti-HDAC3 (1:500, CST, 3949), anti-FLAG (1:3000, Sigma-aldrich, F3165), anti-ACSS2 (1:1000, CST, 3658), anti-LDHA (1:2000, CST, 3582), anti-LDHB (1:5000, Protein Tech,14824-1-AP), anti-VDAC (1:1000, CST, 4661), anti-Lamin B1 (1:1000, CST, 2125), anti-H3 (1:20000, Millipore, 07-690), anti-CBP (1:500, CST, 7389), anti-p300 (1:500, Santa Cruz, sc-48343), Goat anti-Rabbit IgG HRP (1:5000, Thermo Scientific, 31460), and Goat anti-Mouse IgG HRP (1:5000, Thermo Scientific, 62-6520).

### RNA extraction and qPCR analysis

Total RNA was isolated using the RNeasy micro kit (Qiagen, 74034) according to the manufacturer's protocol. cDNA was generated from equal amounts of RNA using iScript cDNA synthesis kit (Bio-rad, 1708891). Quantitative PCR (qPCR) with Power SYBR Green PCR Master Mix (Applied Biosystems, 4367659) was carried out using CFX384 real-time PCR detection system (Bio-rad). The primer sequences are provided in Table S1.

### *In vitro* lactylation assay with recombinant HDACs

*In vitro* lactylation assays were performed as described previously (26). Most *in vitro* reconstitution experiments used recombinant proteins from Cayman: rHDAC1 (10009231), rHDAC2 (36419), and rHDAC3/NCOR2 (10009232). rHDAC2 from BPS Bioscience (50002) was used for Fig. S1, *A* and *B*. We selected vendors based on the availability of recombinant proteins from each company at that time and observed no difference in rHDAC2 acylation capability among the vendors. Recombinant Histone H3.1 (rH3) was from NEB (M2503S). In most experiments, 1 μg rHDAC and/or 1 μg rH3 was incubated in reaction buffer (50 mM Tris-HCl pH 7.5) with sodium L-lactate (71718, Sigma-Aldrich) for the indicated time at 37 °C. Reactions were performed in 8-strip tubes in a thermal cycler (ProFlex PCR system; Applied biosystems) in a final reaction volume of 15 μl. Reactions were stopped by adding 4× Laemmli buffer and subsequent boiling at 95 °C for 5 min or acetic acid (final. 0.15%) for Western blot or mass-spectrometric analysis, respectively.

### LC-MS/MS analysis for *in vitro* acylated histone proteins

#### Derivatization/propionylation

Propionylation of lysine residue was performed using propionic anhydride and isopropanol in a 1:3 ratio. Equal amounts of protein between conditions were adjusted to pH 8 using NH_4_OH before the addition of 10 μL of propionylation reagents to each sample followed by vortexing, pH was again adjusted to eight by adding 5 to 8 μl of NH_4_OH. The reaction was incubated at 37 °C for 15 min and repeated a total of 3 times to ensure complete propionylation.

#### Trypsin proteolysis

Propionylated histones were diluted with 10 μl of 50 mM ammonium bicarbonate buffer and digested using Trypsin at a 1:50 ratio of protein to trypsin, overnight at at 37 °C. Extracts were combined, concentrated to dryness by SpeedVac, reconstituted in 0.1% formic acid and desalted using C18 Stage-Tips. Digested peptides were dried using SpeedVac, and dried peptides were cleaned using C18 Stage-Tips before LC-MS/MS.

#### LC-MS/MS analysis

The C18 cleaned peptides were analyzed on Thermo Scientific Orbitrap Exploris 240 mass spectrometer interfaced with Thermo Scientific UltiMate 3000 HPLC and UHPLC Systems. Peptide digests were reconstituted in 0.1% formic acid and were separated on an analytical column (75 μm × 15 cm) at a flow rate of 300 nl/min using a step gradient of 1% to 25% solvent B (0.1% formic acid in 100% acetonitrile) for the first 50 min and 25% to 30% for next 2 min, 30% to 70% for 2 min, 70% to 1% for next 2 min the total run time was set to 60 min. The mass spectrometer was operated in data-dependent acquisition mode. A survey full-scan MS (from m/z 400–1600) was acquired in the Orbitrap with a resolution of 6000. Data were acquired in topN with 13 dependent scans. Fragmented using normalized collision energy with 37% and detected at a mass resolution of 60,000. Dynamic exclusion was set for 8 s with a 10 ppm mass window.

#### Data analysis

MS/MS searches were carried out using SEQUEST search algorithms against a custom-made database for human histones using Proteome Discoverer (Version 3.0, Thermo Fisher Scientific). The workflow included Spectrum files, Spectrum selector, SEQUEST search nodes, Target decoy PSM validator, IMP-ptmRS, peptide validator, event detector, precursor quantifier. Oxidation of methionine, propionylation at lysine, lactylation at lysine, and *N*-terminal protein acetylation were used as dynamic modifications. MS and MS/MS mass tolerances were set to 10 ppm and 0.02 Da, respectively. Trypsin was specified as protease and a maximum of two missed cleavage was allowed. Target-decoy database searches used for calculation of false discovery rate (FDR) and for peptide identification FDR was set at 1%. Feature mapper and precursor ion quantifier were used for label-free quantification.

### *In vitro* deacetylation assay

FLAG-IPed HDAC (WT and mutants) proteins were transferred to a 96-well half-area assay plate (3994, CORNING), and the buffer was completely removed. Deacetylation assay with fluorescent molecule-conjugated peptide was performed using HDAC Fluorometric Activity Assay Kit (10011563, Cayman), according to the manufacturer’s protocol. The reaction volume was 85 μl, and the deacetylation reaction was performed for 30 min. The fluorescent signals in duplicates were detected by plate readers at excitation = 350 nm and emission = 460 nm (SpectraMax iD5, Molecular Devices). Relative IP 3xFLAG-mHDAC2 amounts were estimated based on in-parallel anti-FLAG Western blot analysis and used for the normalization toward both *in vitro* lysine lactylation activity and *in vitro* deacetylation activity.

### Dialysis following *in vitro* lactylation experiment

*In vitro* lacylation was performed as described in “*In vitro lactylation assay with recombinant HDACs*”, with 2 μg rHDAC, 2 μg rHDAC, and/or 1 mM L-Lactate for 10 min at 37 °C, performed in 8-strip tubes in a thermal cycler (ProFlex PCR system, Applied Biosystems) in a final reaction volume of 30 μl. After the *in vitro* lactylation, four samples (lanes 2–5) were pooled, and 30 uL of the mixture was transferred to Slide-A-Lyzer MINI Dialysis Devices (3.5 K MWCO, 69550; Thermo Scientific). The dialysis devices were submerged in 1.7 ml of 50 mM Tris-HCl pH7.5 buffer, containing either no L-Lactate, 1 mM L-Lactate, or 5 mM L-Lactate, and incubated for 1 h at room temperature (23 °C) with continuous agitation at 300 rpm using a heat block (Thermo Scientific). For a control sample (lane 2), 30 uL mixture within the dialysis device was left for 1 h on a 2 ml tube without any solution, and the reaction was stopped by adding 4× Laemmli buffer and subsequent boiling at 95 °C for 5 min. For dialysis samples (Lanes 3–5), after dialysis at room temperature, samples were transferred to 8-strip tubes and further incubated at 37 °C for 2 h to facilitate enzymatic reactions. Reactions were stopped by adding 4× Laemmli buffer and subsequent boiling at 95 °C for 5 min for Western blot.

### Peptide synthesis and purification

Fmoc-Lys(lac)-OH was synthesized according to previously published procedures (30, 31). H3^6-13^ derived peptides were synthesized by manual Fmoc solid phase peptide chemistry on Rink-Amide MBHA resin. The resin underwent iterative cycles of Fmoc deprotection (20% piperidine in DMF, 30 min), amino acid coupling (4 eq. Fmoc-protected amino acid, 4 eq. HATU in 4% DIPEA in DMF, 1 h) and washing (3 × DMF) until completion of the sequences. The N-terminal Abz group was installed by coupling with 5 eq. Boc-2-Abz-OH, 5 eq. HATU and 10 eq. of DIPEA in DMF for 1 h. Peptides were cleaved from the resin in a mixture of TFA:TIPS:H_2_O at the ratio of 95:2.5:2:5 for 3 h at room temperature. The peptides were precipitated using ice-cold diethyl ether, centrifuged at 4500 rpm at 4 °C for 5 min, redissolved in a mixture of H_2_O and MeCN and purified by semi-preparative reverse phase chromatography eluting with 5 to 95% MeCN in H_2_O containing over a C18 column. Peaks containing peptides were identified by LC–MS, pooled, and concentrated *in vacuo* to yield bright yellow solids. Peptide Sequences: AbzTARKSTGK(Dnp), AbzTARK(Lac)STGK(Dnp).

### HDAC2-catalyzed lactylation kinetics experiments

Recombinantly expressed HDAC2 was commercially sourced from Activemotif (Cat. no 31505). HDAC2-catalyzed reactions were performed in a 300 μl final volume in 50 mM Tris buffer, pH 8.0 containing 10 mM DTT, 150 mM NaCl and 5 mM MgCl_2_. 10 μM of peptide (AbzTARKSTGK(Dnp)) was incubated with 167 nM HDAC2 and varying concentrations of L-lactate (0–5000 μM) for 1 h at 37 °C. Lactylation reactions were quenched by addition of 5 μl of deacetylase inhibitor cocktail (100x in 70% DMSO) and incubated for 5 min. Subsequently, samples containing the lactylated peptides were transferred to 384 well Corning NBS microplates (flat bottom, no lid, low flange, non-binding surface, non-sterile, and black), and trypsin was added to a final concentration of 1 μg/ml to determine the amount of unmodified lysine peptides in the reaction. The cleavage of the lysine-containing Abz/Dnp-substrate was monitored by measuring the fluorescence intensity in a Varioskan LUX microplate reader at emission and excitation wavelengths of 320 and 420 nm. ΔFluorescence values were extracted as the difference between the start and end of the reaction. Triplicate measurements were taken for each data point. The fluorescence data were processed using GraphPad Prism, and for the kinetics measurements, the data were analyzed using nonlinear regression according to Michaelis-Menten kinetics. The data were reported as mean ± SE%.

### *In vitro* lactylation assay with whole cell lysates

For whole cell lysates, HEK293T cells were harvested and washed with PBS. Approximately 1.0 × 10^6^ cells were lysed with 100 μl of Buffer 1 (50 mM Tris-HCl pH 7.5, 150 mM NaCl, 5 mM MgCl2, 0.5 mM ZnCl2, 1 mM DTT, 1xHalt Protease inhibitor). Cell lysates were sonicated using a Branson Sonifier 450 and centrifuged at 14,000 rpm for 5 min to pellet the insoluble proteins. Protein contents in the supernatants (soluble protein fraction) were quantified with the DC Protein Assay Kit (BioRad Laboratories), and 10 μg of the native whole lysates were used for *in vitro* lactylation assays on a 20 μl scale. *In vitro* lactylation assays were performed as described in the above section.

### Subcellular fractionation

Subcellular fractionation experiments were performed using the Kit (Thermo Scientific, 78840) according to the manufacturer’s protocol. HEK293T cells were harvested and washed with PBS. The cell pellet of 1.0 × 10^6^ cells was estimated to correspond to a volume of 10 μl. Protein contents in each compartment were quantified with the DC Protein Assay Kit (BioRad Laboratories), and protein concentrations were adjusted based on the quantification using RIPA buffer. Equal amounts of protein for each fraction were used for subsequent Western blot analysis.

### Lactate quantification

Cells were harvested after trypsinization and quenching with their respective culture supernatants to maintain extracellular lactate concentrations during quenching and cell counting. After counting, cells were washed once with PBS, resuspended with 25 μl metaphosphoric acid (MPA) per 1.0 × 10^∧^6 cells and incubated on ice for 5 min. Following centrifugation at 10,000×*g* for 5 min at 4c, 20 μl of the supernatant was transferred to a new tube and neutralized with 1 μl of potassium carbonate to bring pH to approximately 8.5. The samples were diluted the sample 1:2 with 1× Assay buffer (50 mM potassium phosphate, pH 7.5), and lactate quantification assay was performed using L-Lactate Assay Kit (700510, Cayman) based on LDH catalysis, according to the manufacture’s protocol. The reaction volume was 100 μl, and LDH reaction was performed for 20 min. The fluorescent signals in duplicates were detected by plate reader at excitation = 535 nm and emission = 587 nm (SpectraMax iD5, Molecular Devices). Intracellular lactate levels were quantified based on a lactate standard curve and normalized to cell numbers.

### Sample preparation for Lactyl-CoA quantification

For HDAC inhibitor-treated samples, cells were seeded at 2.5 × 10^6^ cells/well in a 10-cm dish. After 1 day of culture, MS-275 (5 μM) or DMSO was directly added to the culture medium. After 24 h, the culture medium was removed, directly resuspended in 1 ml of 10% (w/v) trichloroacetic acid (TCA), and stored at −80 °C until further processing. Six dishes were used as technical replicates for each condition.

### Liquid chromatography mass spectrometry detection of lactyl-CoA

Acyl-CoAs were analyzed by liquid chromatography-high-resolution mass spectrometry (LC-HRMS) as previously described (17). 50 μl of short-chain acyl-CoA ISTD was added and then cell suspensions were sonicated with 5 × 0.5-s pulses at 50% intensity (Fisherbrand Sonic Dismembrator Model 120 with Qsonica CL-18 sonicator probe). Lysates were centrifuged at 17000*g* for 10 min at 4 °C and clarified lysates were transferred to a deep-well 96-well plate for loading in a Tomtec Quadra4 liquid handling workstation. On the liquid handling workstation, lysates were applied to an Oasis HLB 96-well elution plate (30 mg of sorbent per well) pre-conditioned and equilibrated with 1 ml of methanol and 1 ml of water, respectively. After de-salting with 1 ml of water, acetyl-CoA was eluted into a deep-well 96-well plate using 1 ml of 25 mM ammonium acetate in methanol. Eluent was evaporated dried under nitrogen gas. A 12-point calibration curve of lactyl-CoA from 0.024 to 25 pmol/sample was prepared along with the samples. The dried LC-HRMS samples were resuspended in 50 μl of 5% (w/v) sulfosalicylic acid in water. 5 μl injections of each sample were analyzed *via* LC-HRMS. Samples were analyzed using an Vanquish Duo ultra-high performance liquid chromatograph coupled with a Q Exactive Plus mass spectrometer (Thermo Scientific) as previously described. A modified gradient using solvent A (5 mM ammonium acetate in water), solvent B (5 mM ammonium acetate in 95:5 (v:v) acetonitrile: water) and solvent C (0.1% (v/v) formic acid in 80:20 (v:v) acetonitrile: water). Data was acquired using XCalibur 4.0 (Thermo Scientific) and analyzed using Tracefinder 5.1 (Thermo Scientific).

### Non-enzymatic *in vitro* carboxy-ethyllation with MGO

Bovine serum albumin (BSA, A9647, Sigma-Aldrich) was incubated in 20 mM phosphate buffer (pH 8.5) with or without methylglyoxal (MGO, 1 mM, M0252, Sigma-Aldrich) at 37 °C overnight.

### Statistical analyses

Statistical analyses were performed in GraphPad Prism (version 10) unless otherwise indicated. We used two-tailed unpaired t-tests to calculate statistical differences between two groups. In analyses in which we compared multiple test conditions to a control group, we used 1-way analysis of variance (ANOVA) with *post hoc* two-sided Dunnett's test to calculate statistical differences. For multiple comparisons between all groups, we used 1-way ANOVA with Tukey’s *post hoc* test to calculate statistical differences. Analysis information for each figure is indicated in the respective figure legends. For all experiments, *p* < 0.05 was considered significant. ∗*p* < 0.05, ∗∗*p* < 0.01, ∗∗∗*p* < 0.001, ∗∗∗∗*p* < 0.0001.

## Data availability

All data related to this manuscript are contained within the main text and supplementary figures.

## Supporting information

This article contains supporting information (Supplementary Figures S1-S7 and Table S1).

## Conflict of interest

The authors declare that they do not have any conflicts of interest with the content of this article.
