# Supplementary material for: Class I histone deacetylases catalyze lysine lactylation[image]
Source: J Biol Chem. 2025 Aug 18;301(10):110602. doi: 10.1016/j.jbc.2025.110602 (PMC12624779; doi:10.1016/j.jbc.2025.110602)
Supplement: Supplementary Figures S1-S7 and Table S1 [file mmc1.docx]

Class I histone deacetylases catalyze lysine lactylation

Michelangelo B. Gozatti^1,Ɨ^, Jordi C. J. Hintzen^2,Ɨ^, Isha Sharma^1,Ɨ^, Mohd. Altaf Najar^2^, Takeshi Tsusaka^1^ Mariola M. Marcinkiewicz^3^, Claudia Veronica Da Silva Crispim^3^, Nathaniel W. Snyder^3^, George M. Burslem^2,4,*^, Emily L. Goldberg^1,5,*^

**Supplemental Information**

This file contains:

Supplementary Figures S1-7

Supplementary Table S1

**
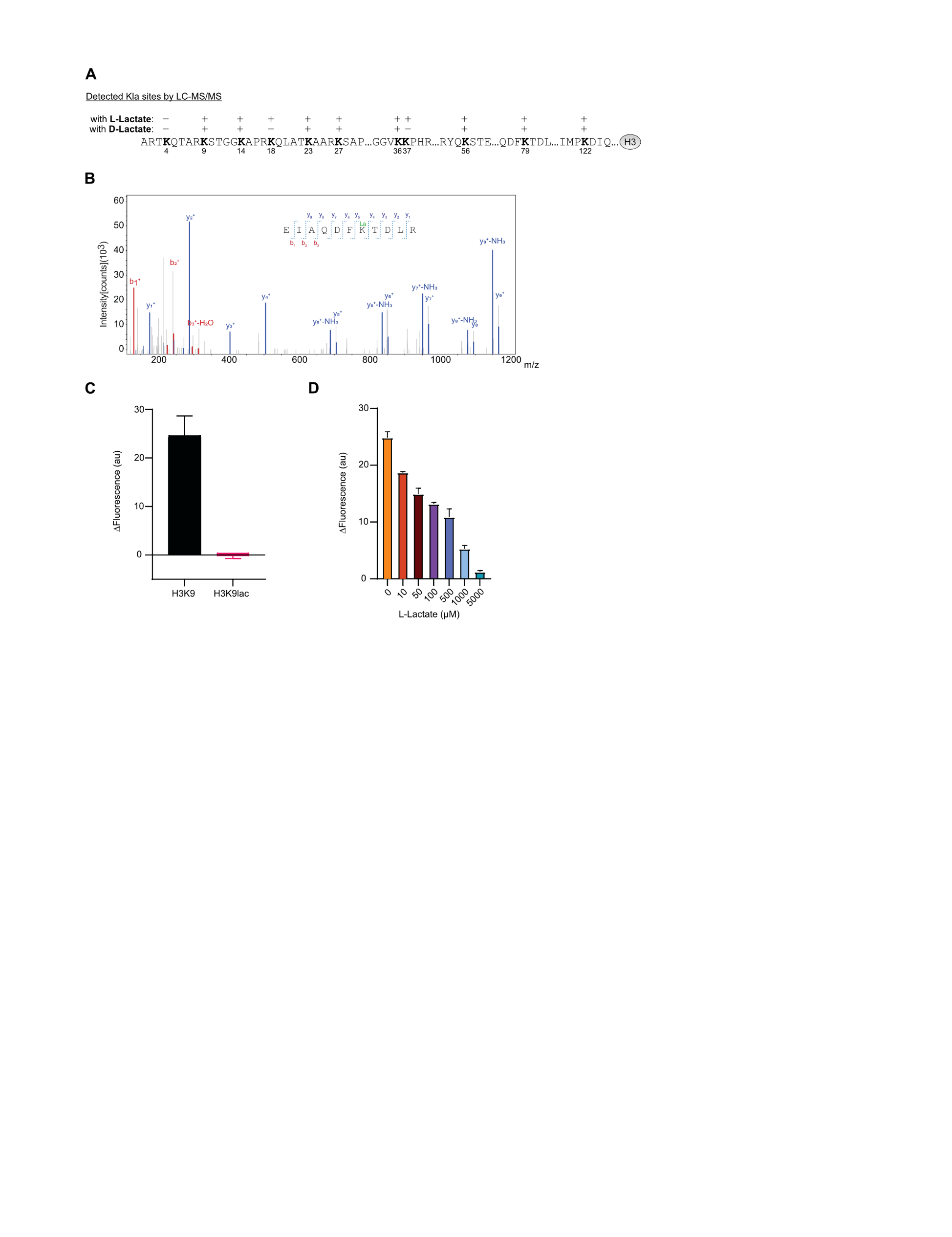
**

**Figure S1. Further analyses of in vitro lysine lactylation by HDAC2**

(**A-B**) LC-MS/MS analysis of H3 following *in vitro* lysine lactylation using ʟ-Lactate or ᴅ-Lactate. (A) Kla-modified sites detected on H3 are indicated with “+”. (B) MS/MS-spectra for the indicated peptide with detected y and b ions. (**C-D**) Control experiments for measuring HDAC-catalyzed Kla formation in a lysine protection assay. (C) The change in fluorescence (ΔFluorescence, arbitrary unit) is shown for synthesized unmodified and lactylated H3K9 peptides. (D) ΔFluorescence (arbitrary unit) after 1 hour incubation of HDAC2 and unmodified H3K9 peptide with the indicated concentrations of ʟ-Lactate. Data represent mean ± SD of three technical replicates from a single experiment.

**
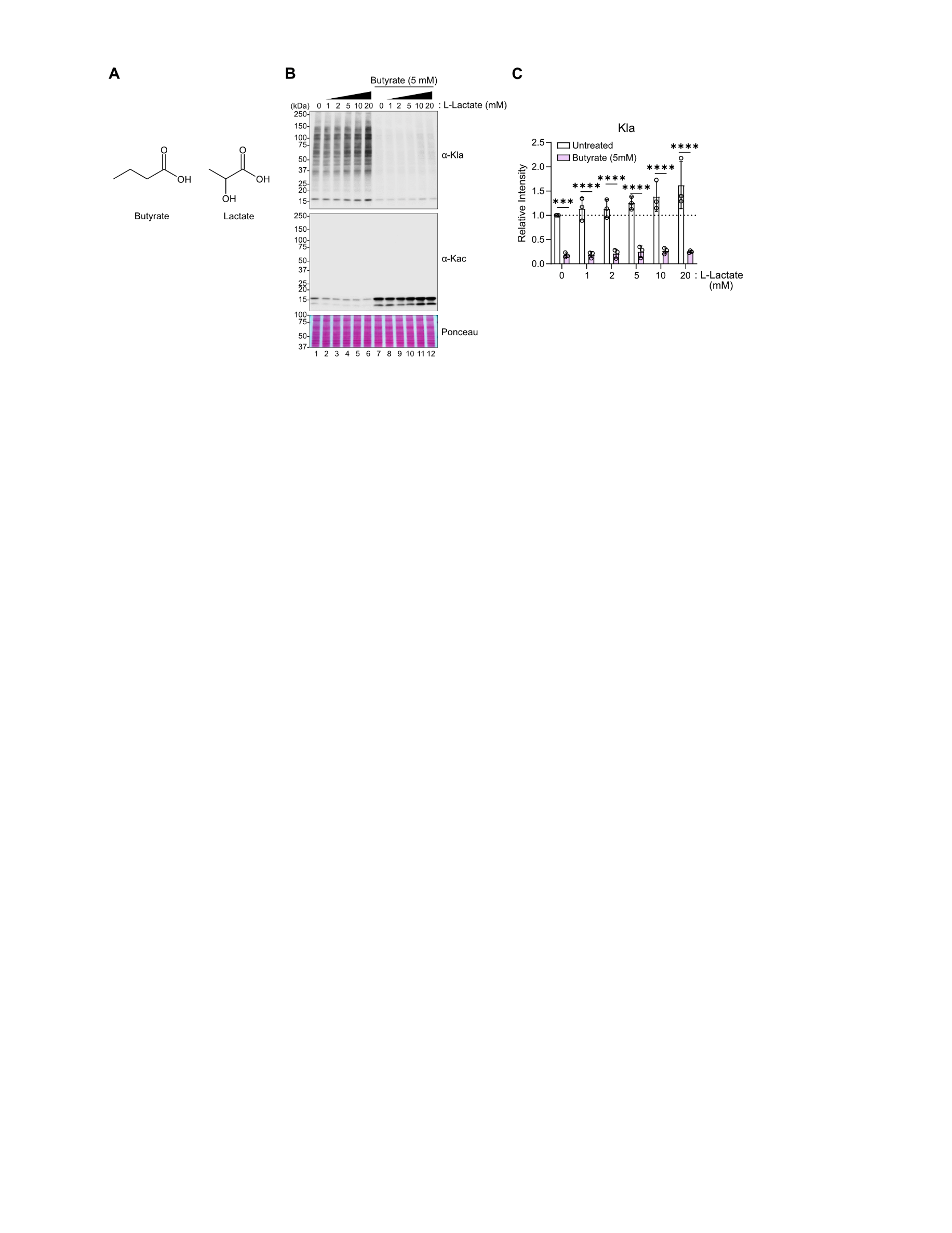
**

**Figure S2. Butyrate, a known histone deacetylation inhibitor, reduces Kla levels**

(**A**) Chemical structures of similar carboxylic acids, butyrate and lactate. (**B-C**) HEK293T cells were treated with ʟ-Lactate and/or butyrate at the indicated concentrations for 24 hours. (B) Representative western blots showing anti-Kla and anti-Kac signals. (C) Quantification of Kla levels relative to untreated (0mM Lactate). Signals were normalized to ponceau S staining. Data represent mean ± SEM from three independent experiments. Each symbol represents an individual experiment. Statistical significance was determined by 2-way ANOVA with Sidak’s test for multiple comparisons to compare with or without butyrate treatment. ***p<0.001, ****p<0.0001

**
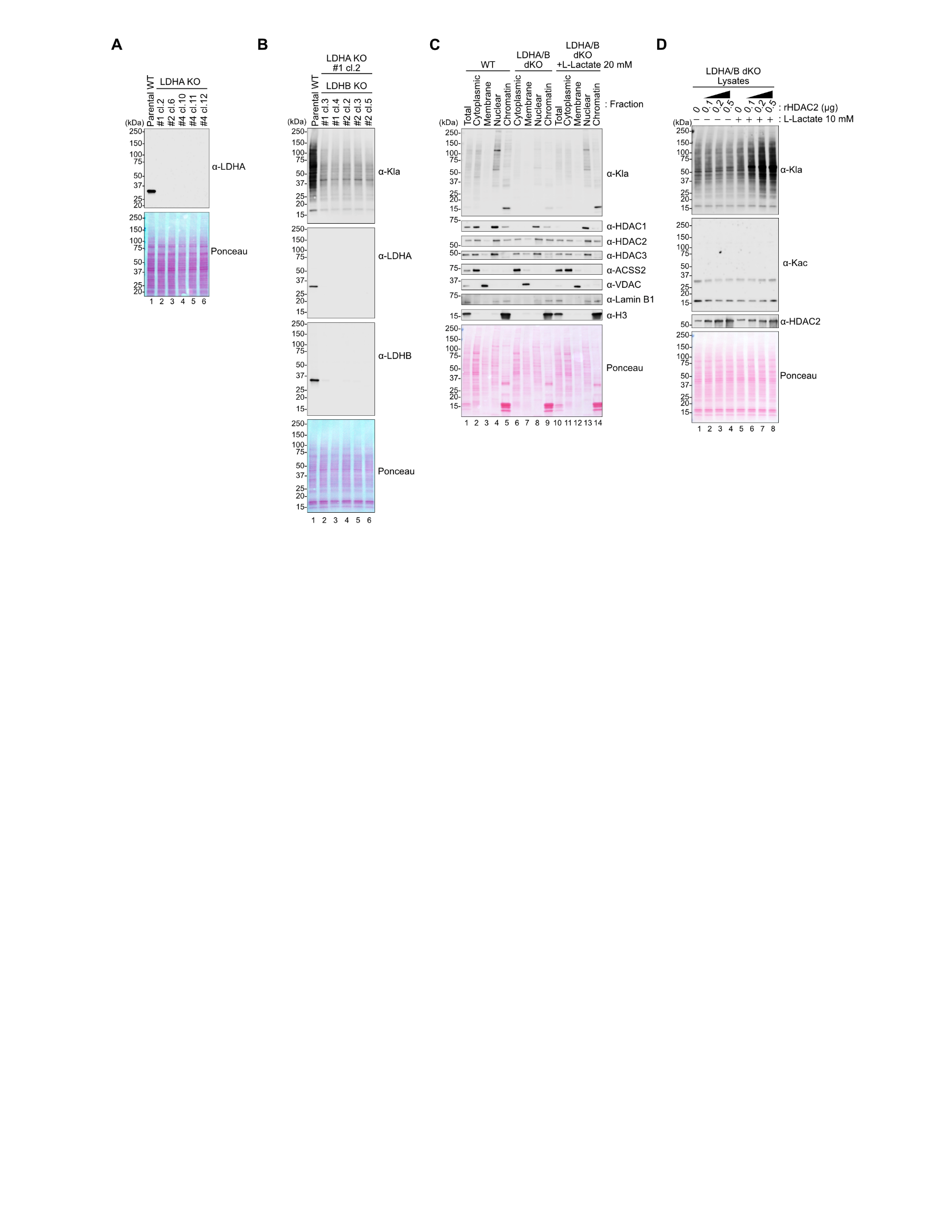
**

**Figure S3. Establishment of LDHA/B dKO HEK293T cells**

(**A**) Cas9-expressing HEK293T cells, labeled as “Parental WT” were transfected with gRNA targeting the *LDHA* gene. After clonal isolation, the deletion of LDHA in each clone was verified by western blot. (**B**) Clone #1 cl.2 (LDHA KO) cells were further transfected with gRNA targeting the *LDHB* gene. After clonal isolation, the deletion of LDHB in each clone was verified by western blot. Clone #1 cl.4 (LDHA/B dKO) was established as a stable line and used for subsequent experiments. Stable lines were always compared to the parental WT control line. (**C**) Cell fractionation was performed using WT and LDHA/B dKO HEK293T cells. LDHA/B dKO HEK293T cells were treated with ʟ-Lactate (20 mM) for 24 hours. Representative western blots for indicated targets are shown, from three independent experiments. Antibodies against ACSS2, VDAC, Lamin B1, and H3 were used as markers for the cytoplasmic, mitochondrial (membrane), nuclear, and chromatin fractions, respectively. (**D**) Whole-cell lysates from LDHA/B dKO cells were used in an *in vitro* lysine lactylation assay with the indicated amounts of rHDAC2 in the presence of ʟ-Lactate (10mM). The reaction was performed at 37°C for 30 min. Representative western blots from two independent experiments are shown.

**
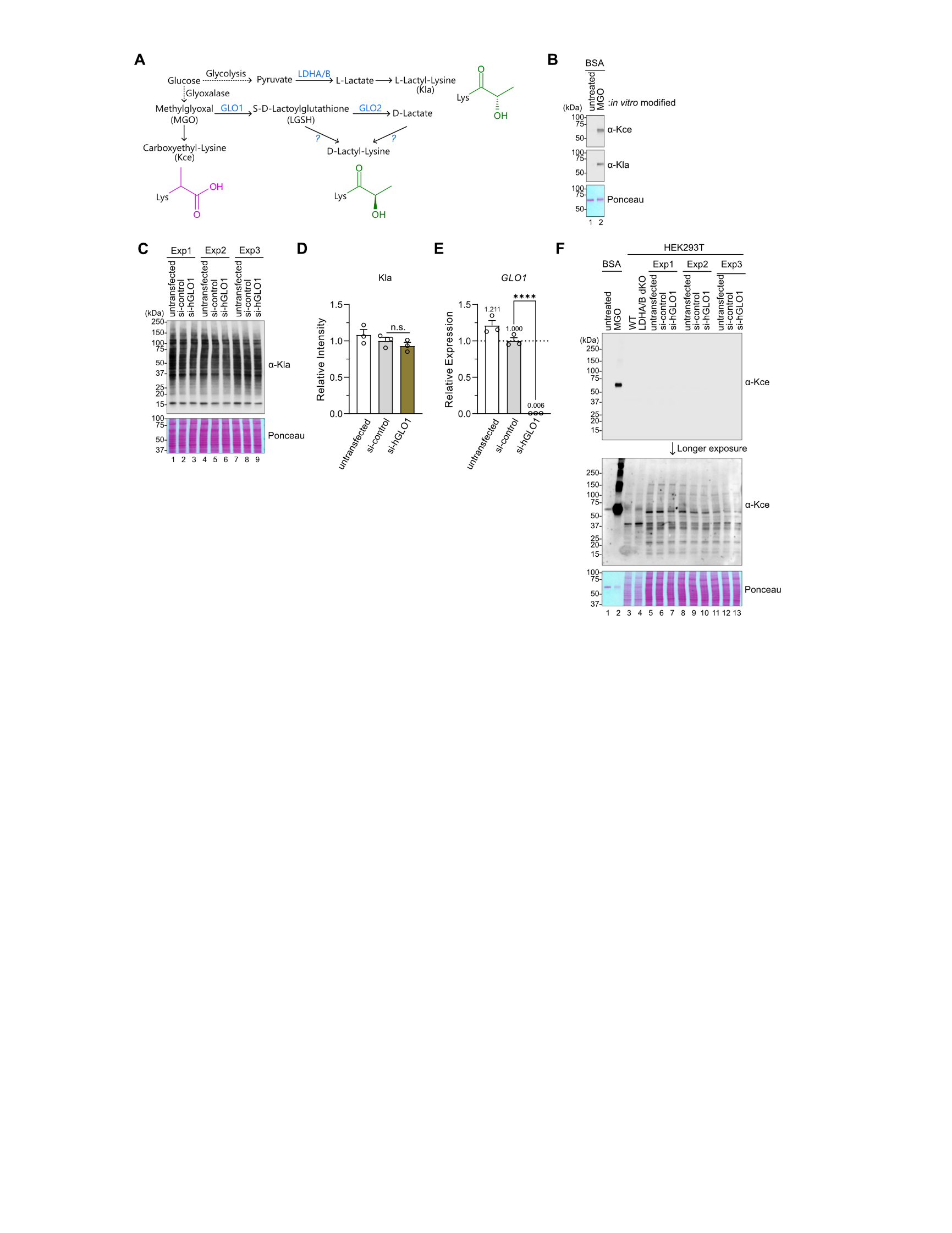
**

**Figure S4. Kla antibody detection is not confounded by intracellular Kce modification**

(**A**) Schematic of metabolic pathways leading to lysine ʟ-Lactylation (Kla), lysine ᴅ-lactylation, and lysine carboxyethylation (Kce). (**B**) Western blot of BSA (bovine serum albumin) incubated with MGO (methylglyoxal), which spontaneously produces Kce modification. (**C-F**) HEK293T cells were transfected with siRNA targeting *GLO1* to assess the potential interference of Kce and ᴅ-lactyl-lysine with anti-Kla antibody recognition. (C) Western blot showing anti-Kla signals from all three independent experiments. (D) Quantification of Kla levels relative to si-control, normalized to ponceau S staining. (E) Quantification of *GLO1* mRNA expression relative to si-control and normalized to *RPL13A* expression. Data represent mean ± SEM from three independent experiments. Each symbol represents an individual experiment. Statistical significance was determined by unpaired t-test. (F) Western blots showing anti-Kce signals from samples in panel “C”, alongside positive and negative controls for Kce and Kla detection (WT and LDHA/B dKO samples used in Fig. 3B). The MGO-modified BSA was freshly prepared. ****p<0.0001, n.s., not significant.

**
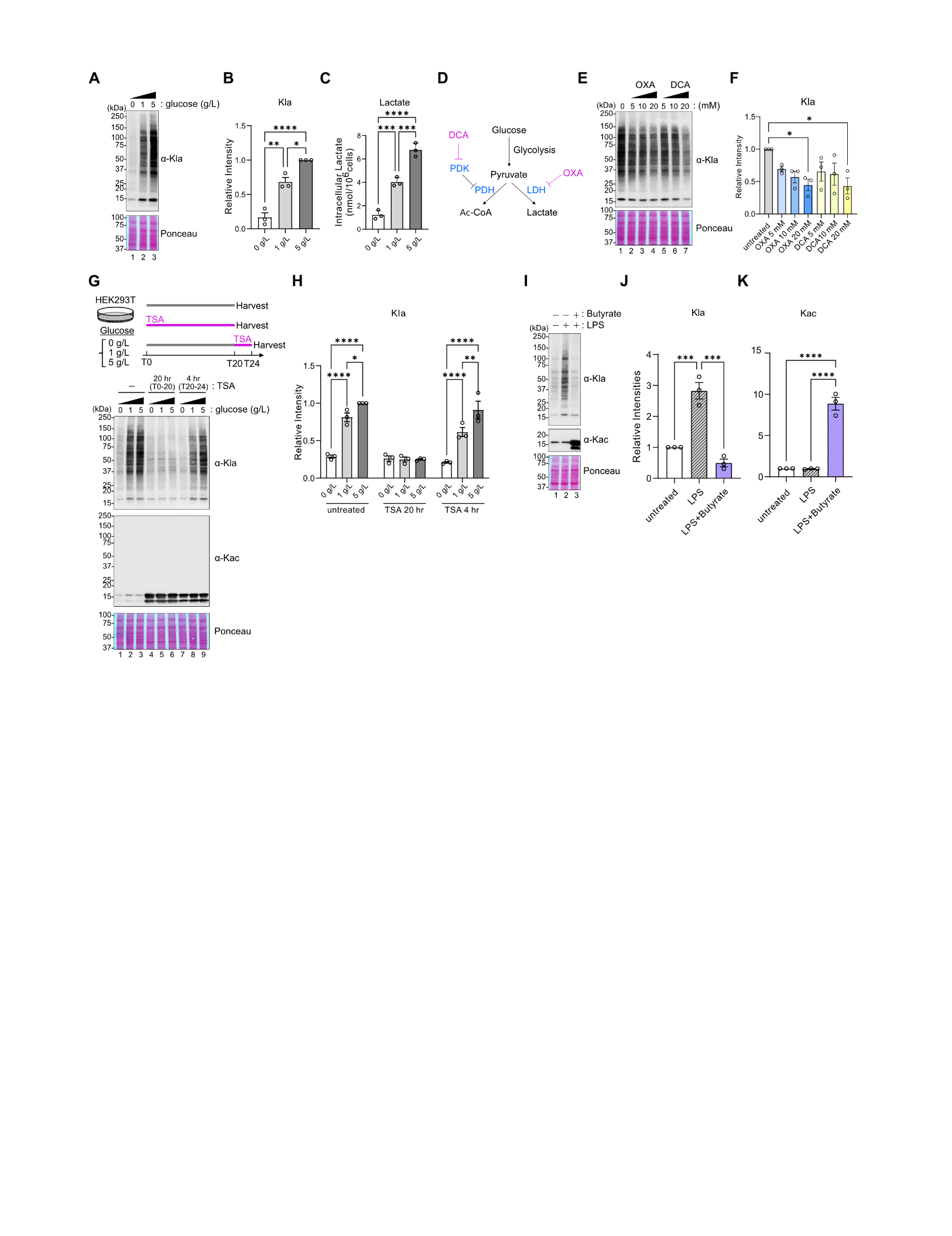
**

**Figure S5. Kla formation is regulated by glucose metabolism**

(**A-C**) WT HEK293T cells were cultured in medium containing 0, 1, or 5 g/L glucose for 24 hours. (A) Representative western blots showing anti-Kla signals. (B) Quantification of Kla levels relative to the 5 g/L glucose condition. Signals were normalized to ponceau S staining. Data represent mean ± SEM from three independent experiments. Each symbol represents an independent experiment. (C) Quantification of intracellular lactate concentrations. Data represent mean ± SD from three technical replicates from a single experiment. Each symbol represents a technical replicate. Statistical significance was determined by one-way ANOVA followed by Tukey’s correction for multiple comparisons. (**D**) Schematic of metabolic pathways and inhibitors used in this figure. DCA (Dichloroacetic acid), OXA (Oxaloacetate), LDH (Lactate dehydrogenase), PDH (Pyruvate dehydrogenase), PDK (Pyruvate dehydrogenase kinase). (**E-F**) HEK293T cells were treated with OXA or DCA at the indicated concentrations for 24 hours. (E) Representative western blot showing anti-Kla signals. (F) Quantification of Kla levels relative to untreated and normalized to ponceau S staining. Data represent mean ± SEM from three independent experiments. Each symbol represents an individual experiment. Statistical significance was determined by one-way ANOVA followed by Dunnett’s correction for multiple comparisons. (**G-H**) WT HEK293T cells were cultured in medium containing 0, 1, or 5 g/L glucose and treated with TSA (1 µM) at the indicated time points. (G) Representative western blots showing anti-Kla and anti-Kac signals. (H) Quantification of Kla levels relative to untreated 5 g/L glucose condition. Signals were normalized to ponceau S staining. Data represent mean ± SEM from three independent experiments. Statistical significance was determined by one-way ANOVA followed by Tukey’s correction for multiple comparisons. Each symbol represents an independent experiment. (**I-K**) Bone-marrow derived macrophages (BMDM) were stimulated with LPS (1 µg/mL) with or without butyrate (2 mM) for 24 hours. (I) Representative western blots showing anti-Kla and anti-Kac signals. (J) Quantification of Kla levels relative to untreated BMDM. (K) Quantification of Kac levels relative to untreated. Signals were normalized to ponceau S staining. Data represent mean ± SEM from three biological replicates of a single experiment. Each symbol represents a biological replicate. Statistical significance was determined by one-way ANOVA followed by Tukey’s correction for multiple comparisons. *p<0.05, **p<0.01, ***p<0.001, ****p<0.0001

**
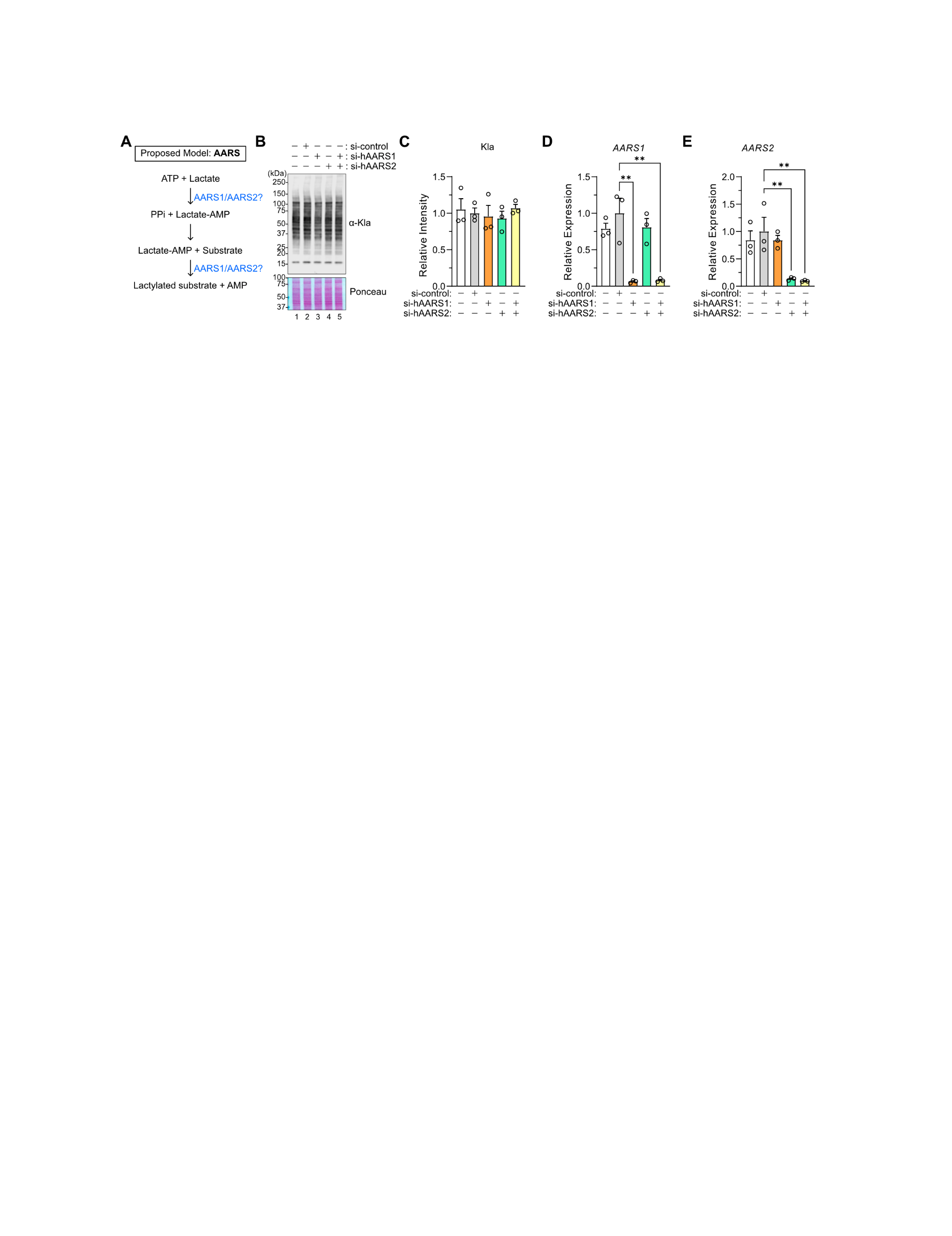
**

**Figure S6. Role of AARS enzymes in basal Kla in HEK293T cells**

(**A**) Proposed model of AARS enzyme-dependent lysine lactylation. (**B-E**) HEK293T cells were transfected with siRNA targeting the indicated genes to assess their role in Kla formation. (B) Representative western blot showing anti-Kla signals. (C) Quantification of Kla levels relative to si-control, normalized to ponceau S staining. (D) mRNA expression of *AARS1* relative to si-control and normalized to *RPL13A*. (E) mRNA expression of *AARS2* relative to si-control and normalized to *RPL13A*. For (C-E) data represent mean ± SEM from three independent experiments and each symbol represents an individual experiment. Statistical significance was determined by one-way ANOVA followed by Dunnett’s correction for multiple comparisons. **p<0.01.

**
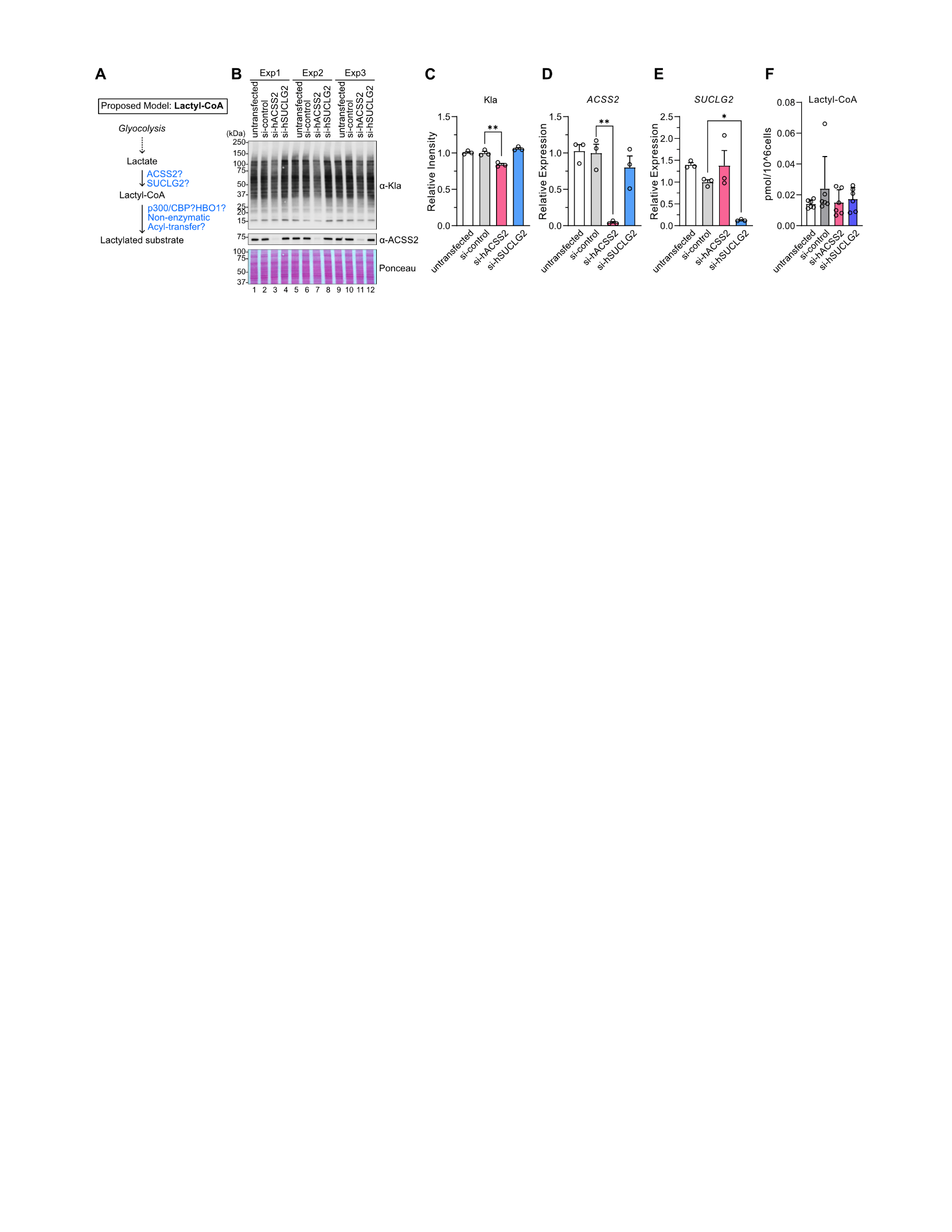
**

**Figure S7. Role of putative lactyl-CoA synthetases in** **Kla in HEK293T cells**

(**A**) Proposed models of enzymatic lactyl-CoA-dependent lysine lactylation. (**B-F**) HEK293T cells were transfected with siRNA targeting *ACSS2* and *SUCLG2* to assess their roles in Kla formation. (B) Western blots for indicated targets from three independent experiments. (C) Quantification of Kla levels relative to si-control. Signals were normalized to ponceau S staining. (D) mRNA expression of *ACSS2* relative to si-control and normalized to *RPL13A*. (E) mRNA expression of *SUCLG2* relative to si-control and normalized to *RPL13A*. Data represent mean ± SEM from three independent experiments. Each symbol represents an individual experiment. Statistical significance was determined by one-way ANOVA followed by Dunnett’s correction for multiple comparisons. (**F**) Quantification of intracellular lactyl-CoA concentrations in HEK293T cells transfected with siRNA targeting the indicated genes. Data represent mean ± SD of six technical replicates, pooled from two independent experiments. Each symbol represents a technical replicate. *p<0.05, **p<0.01

| Oligo Name | Oligo sequence (5' 🡪 3') |
| --- | --- |
| hACSS2-F | TGCACGGCAGACATTGGTTG |
| hACSS2-R | GGGAATCCCCTCAAACAAAACAC |
| hGLO1-F | GTCCCGTCGTCTGTGATACTG |
| hGLO1-R | GTAGCATGGTCTGCTGCAATA |
| hAARS-F | TGTGCGAGTCGTCTCCATT |
| hAARS-R | TCTTCCGTCACGATCACAAA |
| hAARS2-F | GCTGCTCAACGCCATAC |
| hAARS2-R | CACTGAGTGTGCGGATGTG |
| hSUCLG2-F | CTGCCGGGAGCTAGAACTCA |
| hSUCLG2-R | GCGACGTTCTCTTGGATACC |
| hHBO1-F | CTGACAAGCGAGTATGACTTGGA |
| hHBO1-R | TTGCTTCCCTCTGTGATTTGG |

Table S1. Oligonucleotide sequences used for qPCR analysis.
